# "The Hospital" Nursing Mirror

**Published:** 1896-12-19

**Authors:** 


"The Hospital" Nursing Mirror.

Being the Nursing Section of "The Hospital."

[Contributions for this Section of "The Hospital" should be addressed to the Editor, The Hospital, 28 & 29, Southampton Street, Strand, London, W.C., and should have the word "Nursing" plainly written in left-hand top corner of the envelope.]

### News from the Nursing World.

### A HAPPY CHRISTMAS.

A Happy Christmas and a Prosperous New Year to our readers, one and all! Never before in the history of The Hospital has there been a year during which we have received so many pleasant assurances of appreciation from correspondents in all parts of the world, or so many congratulations on present progress and good wishes for future success and prosperity, and we gladly take this opportunity of replying with most cordial greetings for Christmas and the coming New Year. It is pleasant to know that the weekly arrival of The Hospital is looked for with eager interest at home and abroad, and that it serves as sometimes the only medium of communication between far-away friends in the hospital world. "In The Hospital," said a nurse the other day, "I see what all my friends and acquaintance of my training days are doing, and can follow the work of many of whom otherwise one would lose sight." We are glad to hear it.

### THE ROYAL NATIONAL PENSION FUND FOR NURSES.

We are requested to ask all nurses to make arrangements so that the collecting books on behalf of the Benevolent Fund special appeal may be received at the office, 28, Finsbury Pavement, London, E.C., by the first post on the morning of Wednesday the 23rd inst., whether any money has been collected or not. It is essential that all who have books shall make a point of returning them by the time specified. In this connection it is interesting to report that a book was received on Saturday from a nurse in South Africa containing subscriptions amounting to £7 6s. When we state that the books were only posted in London on October 24th, it will be seen that this is distinctly a record. Amongst the subscribers is the name of the Right Hon. Cecil Rhodes, £1 1s.

### GUY'S HOSPITAL.

Great changes in the nursing department of Guy's Hospital are now being organised. A nursing home of ample proportions, and replete with every modern improvement, is about to be built. The staff will probably be considerably increased in number, and the nurses will gain much which will make for health and comfort in many ways. Miss Nott-Bower is to be congratulated on having secured the co-operation of Miss Esther H. Young, whose splendid work at Addenbrooke's Hospital marks her out as one of the best of organisers and the deservedly popular head of a large nursing staff. Miss Young's nursing qualifications and gifts are exceptionally high, and her kindly consideration for everybody secures the maximum of efficiency, for love is still a stronger force than any other known to man.

### "TRUTH" DOLL AND TOY SHOW.

On Wednesday and Thursday in this week the Albert Hall was transformed, for the fifth successive year, into a perfect children's fairyland. Seventeen years ago the first "Truth" Toy Show was held in two small rooms in the City; now the great arena of the Albert Hall can hardly contain the thousands of dolls and toys of all descriptions which will be sent out during the next few days to the hospitals and workhouses of London. Nearly 200 institutions will share in this year's distribution, and the total number of toys reaches almost 30,000, including 4,000 dolls. Some of these latter were quite dreams of beauty in their dainty attire; among them were also several in nurses' garb, including one in the picturesque dress of an army sister. Guarded by a stalwart policeman was a glass case containing the 11,000 new sixpences annually sent by an anonymous donor for distribution amongst workhouse children, and, as usual, Mr. Tom Smith sent many thousands of crackers.

### A GOOD IDEA.

The nurses of St. Mary's Hospital, Paddington, have hit upon an excellent way to celebrate Christmas time this year for their patients, only the cost of carrying it out ought to come from other pockets than those of the nurses! They are going to hire a piano for a week for each ward, so that a series of pleasant concerts may delight the patients throughout the week, at the most convenient hour each day. Kind friends are wanted to help by undertaking to provide programmes for the various days, help which should be very easily secured, for it is but a small thing to do after all, only necessitating a little trouble in finding friends with musical gifts to give their services, and a little sacrifice of leisure. We hope Miss Medill may receive plenty of offers of assistance before Christmas is actually upon us.

### CHRISTMAS FESTIVITIES.

All notices of Christmas festivities and entertainments which our readers wish to have inserted in The Hospital should be sent to the office as promptly as possible. We shall be very glad to find space for such contributions as far as may be, but they are only suitable just for the present season, and our numerous correspondents must send in their reports directly their special rejoicings are over or they will be too late for publication.

### SHORT ITEMS.

The nuns at St. Vincent's Hospital, Dublin, have recently acquired two adjoining houses for the reception of private patients. The nursing school at this hospital has prospered well, and the nuns have now a qualified staff to send out to private cases.—Princess Christian has accepted a copy of "A Text Book of Nursing," by Miss Clara Weeks-Shaw, edited by Mr. William Radford, house surgeon at the Poplar Hospital. The book is dedicated to the Princess.—The National Sunday League, a Sunday or two ago, provided concerts for the inmates of Holborn Union and St. Olave's Union workhouses, and the music gave great pleasure to the large audiences who gathered to hear it.

### Christmas throughout the World.

### Christmas in the Dublin Hospitals.

FROM A CORRESPONDENT.

THE most attractive of our Dublin hospitals, to visitors, is undoubtedly the Rotunda, for the good taste exhibited by its generous founder, Dr. Mosse, and the architectural style of the period during which it was built (1750) accord with the revival of last centuryism which holds the public eye and fancy at the present day. The late master, Dr. W. J. Smyly, and his wife are so well known in Dublin society that they attracted a widespread interest to the hospital during their seven years' residence there, their large and influential circle of friends contributing not only to the general funds but sending at Christmas time special evidences of goodwill in the shape of turkeys, country produce, and a wealth of glossy evergreens and flowering shrubs wherewith to decorate the quaint old wards. Miss Hampson, who was placed at the head of the nursing school on its foundation six years ago, was trained at the Nightingale School at St. Thomas's, from whence she brought the kindly custom of singing carols to the patients on Christmas Eve. Her choir is composed entirely of nurses, and has been trained each year by Miss Margaret St. Clair, superintendent of the St. Laurance's Home for the Queen's Nurses, a former fellow student of Miss Hampson. The governors of the Rotunda and other friends are invited to attend, and are entertained to tea in the board-room, a wainscotted apartment of dignified proportions hung with oil portraits. This is the first Christmas of the new master's (Dr. Purefoy) administration, and the last of Miss Hampson's as superintendent; but no doubt similar hospitality will be continued to patients and guests alike—that wonder of wonders, "The Incubator," again explained to open-eyed outsiders by its high priestess, Nurse Anderson, and the "Venite Laudamus," heard swelling from the fine old stone entrance hall, as the choir starts on its tuneful round, up the grand staircase, past the closed doors of the beautiful chapel decorated by the Franchini, along the first groined corridor of the old Maternity, on to the new wing, where one enters on a scene of all that is most modern in hospital management—red-tiled passages, the hydraulic elevator, wards as wide as many a village street, palms growing healthily, and patients recovering satisfactorily after serious operations.

In all our Irish "medical houses" roast beef and plum pudding are supplied for all who are permitted by the doctors to partake of this regulation cheer, and invariably there is a "Fairy Tree" for the children; then at most places the nurses and students organise a concert through the wards, or lantern show in the theatre, to which friends and convalescents are bidden. What a hero is the student who can recite a stirring battle piece or romantic tale; what a delight to many is the junior resident who owns a banjo, and can twang it in accompaniment to "Tim Finnigan's Wake," in the big men's ward, or "Love was Once a Little Boy" to the women patients in their special department.

At the old, French château-like pile erected by those truly heavenly twins, Dr. and Miss Grizzel Steevens, in good Queen Anne's time, the staff generally give a very fine concert indeed, because they possess two finished musicians in Drs. Richard Hayes and Henry Tweedy; and, as the hospital keeps a ward at the service of the Royal Irish Constabulary, these gallant fellows bring their celebrated band into action both as soloists and for the nurses' dance, which follows.

At the Eye and Ear Infirmary Miss Clara Lee holds a distribution of welcome and substantial clothing among her patients on Christmas morning, and later, conducts most of them to the high tea, concert, and tree at good old Sir Paddy Dun's, where her fellow pupil of Bart's, Margaret Huxley, one of the ablest of nurses, has been superintendent these twelve years. The Home for Incurables at Donnybrook is quite a jovial place during the holidays, for many of the chronics there have been in for half a lifetime, and hold small levées of visitors. The Queen's Nurses carry their good things with them about their work; clothing for women and children, blankets, soup, and orders for coals, for which a certain great lady gives them *carte blanche*.

At the Military Hospital there is no official recognition of the great Festival (which is to be regretted), but Superintendent Sister Dew and her nurses club together to give their poor fellows a nice tea.

The sisters of charity at St. Vincent's Hospital provide very good cheer for their patients, and a tree for the children. At Our Lady's Hospital for the Dying, Harold's Cross, there can be no rejoicing, but some of the patients are carried to the organ gallery of the chapel to hear the singing Mass, which alone celebrates the great Festival there. The Hospice is made the brightest of all conventual houses, and the nuns pursue the even tenor of their quiet, unselfish lives on feast and fast day alike, praying for the passing soul as it takes wing and performing the last offices for the dead.

### Christmas in Military Hospitals in India.

BY AN ARMY NURSE.

AMIDST the gorgeous festivities of an English Christmas it may interest readers of THE HOSPITAL to know how it has been kept in other lands. I shall first describe how Christmas was kept in an Indian hospital, where there were no nursing sisters. The soldiers tell the tale to this day. The patients had it all their own way; they broke almost every chair in the hospital and several other things—the iron-clamped invalid chairs which tell the tale, I have no doubt, may yet be seen. Thirty of the soldier patients were made prisoners and put under arrest, but not till the medical warrant officer on duty had been severely mauled. Beer was conveyed surreptitiously into hospital in the water-bottles or sarahis, and many indigestible articles, such as ham and plum duff.

Little is done for a sick soldier in hospital at Christmas-time, but the small efforts made are much appreciated. Christmas cards are always welcomed.

I'll tell you of two Christmases I witnessed when in India in military hospitals. I was alone one year at a station, and had few acquaintances, so was left to my own resources. The senior medical officer gave me leave to have a tea in one of the special wards, stipulating that I submitted the menu to him. The orderlies and I decorated the wards with bunting, large bunches of Bourgainvillias, pointseltias, and other tropical flowers; the patients in their beds produced impossible paper flowers of every shape, shade, and hue, and gorgeous coloured paper festoons; they called them cat's cradles; I sacrificed my artistic tastes to the wishes and pleasure of the majority, and displayed them boldly with nature's trophies, where they stood out against the bunting. They thought it all most beautiful, especially the one or two mottoes, in which, invariably, in "Happy" the p's turned the wrong way, and the small letters were often larger than the capitals.

The bedside tables were converted into tea-tables, and covered with real British damask tablecloths. The dining tables, and a round table in the centre, were set out for men from other wards, and the few in mine who could sit up. We made it look as English and home-like as possible; in the place of the huge regulation mugs we had real china cups and saucers, teaspoons, and the tea made in brown teapots, and the best tea, too, that could be bought; very different from the boiled compound that usually bears that name in hospital. They enjoyed the clatter of the spoons, cups and saucers as much as anything, so different from the thud of a mug coming down on the table. One poor lad was so excited watching the preparations, asking us what was on each plate, if he might have a big cup and saucer, and if he might milk and sugar his own tea, &c., that he dropped off at last from sheer exhaustion, and slept for some hours. He was none the worse when he woke up, and his share had been saved for him. We had a bran-pie, with at least two presents for each in. For tea there were jellies, blanc manges, jam without seeds or stones, Madeira and sponge cakes, thin bread and butter, and, for a very limited few, tongue sandwiches. Our pleasures, you see, were very simple, but all the same it was a red-letter day to many, and was talked of for many a week, and my successors were told of it as a gentle reminder the following December, and they responded.

My last Christmas in India my patients were too ill for teas and festivities, but the little that was done made them very happy.

I was lucky enough to have some holly and mistletoe from the hills sent to me, and the Agent for Moore and Co., too, gave me a hamperfull as well. These are by no means common in India, and difficult and expensive to get. Three ladies sent bundles of Christmas cards; these I divided out equally without favour, giving two to each. I tied up evergreens into bunches, allowing one for each man, and putting holly and mistletoe into each. Luckily I was then on night duty, so the orderlies and I stole quietly round in the night and tied them behind each man's bed, placing the cards on their pillows. One or two weary ones who were half asleep and had not seen holly or mistletoe for many years thought they were dreaming and back at home—that it could not be real. One said, "This is the first time I ever heard of Santa Claus in India; I'd have put up my sock if I had known."

In the morning as they awoke their surprise and pleasure were almost childish; they gloated ever their cards and the most sentimental were the most admired and were put aside with care to be sent home. Some begged that little bits of mistletoe might be snipped off for them, and they lay in rapture gazing at the decorations opposite them, too weak to look up at their own. They cracked their feeble jokes, such as "Plenty of mistletoe and never a girl," and were hearty in all their "Merry Christmases." Our only married man, an old service one, presented his wife with ineffable pride with a button-hole and the cards when she came to see him after church; and those on port and brandy drank "Merry Chri tmases" to the others. And so we spent a quiet day. The Wesleyan minister and his daughters afterwards came round with more cards and flowers, the young faces of the latter also giving pleasure to our patients.

### My First Christmas in the United States.

By an English Matron.

A hospital Christmas has comparatively little significance in the United States, where, as in France and Russia, the New Year is a much more important function.

But being English, and the matron of a charming little hospital in Pennsylvania, under the inspiration of that home-sickness natural to one spending a first Christmas in a new country, I determined that my American patients and staff should spend their Christmas in the good old-fashioned style.

At first the suggestion "scared"—to use an Americanism—some of the Quaker members of the committee, in whose eyes holly berries savoured suspiciously of "Popery" and the scarlet woman, and Christmas carols of Rome and heresy. But persuasion and the charm of novelty had their effect, and I was given a cordial permission to "go straight ahead."

The hospital staff were quite excited and anxious to keep Christmas in feudal fashion, for it is really difficult to persuade the untravelled American that we have abolished curfews and drawbridges. Their notions of Christmas merry-making were limited to the artistic dressing of a gigantic and present-laden tree. And even this looked very foreign when it was finished, for, be it remembered, Christmas customs were imported to the States mainly from Scandinavia and the Fatherland, and I couldn't get the German look out of that tree. A visit to the market revealed the scarcity of evergreens with which to make our wards beautiful, holly with berries being quite an expensive luxury, while a bit of "English mistletoe" made extravagant inroads on the "greenbacks"—the dollar bills—in my purse. But berry-laden holly and mistletoe we were determined to have, and, Christmas coming but once a year, reconciled us to present bankruptcy.

The first festive preparations brought a requisition from the nurses. "Might they be excused from wearing uniform on Christmas Day?" for the American nurse is apt to feel she can never have quite such "a good time" in hospital garb as in the dress of her own devices. On the eventful day the head nurse of the children's ward came out resplendent in a maroon silk gown, whose hem satisfactorily swept the ward floor. Her hair was dressed "à la Jap."—a very popular style in the States—and she was perfectly happy and quite unconscious that she looked infinitely prettier in the frilled white cap of every day, whose fashion was imported from London, and the regulation cotton gown with lilac "sprigs."

On reflection, I decided to make the puddings myself, the Irish cook not being absolutely reliable when it came to a question of sustaining the reputation of so national a dish. The dispenser was vastly dismayed when he discovered the amount of "best brandy" it takes to compound a genuine English pudding—to say nothing of the accompanying sauce! A few new sixpences were added to the original recipe to emphasize the excellences of the Christmas pudding institution. And the "stir-for-luck" was indulged in by the whole staff, including the house surgeon, all being amused by the novelty of the idea.

Snap-dragons fell exeedingly flat, and this was the only failure to record. The children were frightened, and the nurses said "they didn't mind burning their fingers and eating raw raisins just once to see what it felt like, but they guessed it wasn't good enough to repeat."

For weeks we were engaged in teaching Christmas carols to the children, and they really gave a most creditable performance on the eventful day when a procession of the managers made rounds with the matron wishing the patients a happy Christmas. But a small boy, one of the sweetest of the carol-singers, somewhat spoiled the gravity of the situation by inquiring, with a bland countenance, "Say, Mr. Chair, do you boss the matron, or does she boss you?"

The Chairman was equal to the occasion. "I guess," he said, reflectively, but with confidence, "I guess the matron bosses me."

The Quaker element on the board, whose identity was easily marked by the fact that their picturesque, broad-brimmed hats remained on their heads during our progress, gave never a smile. But the Gentiles enjoyed the incident, and declared it had been rehearsed with the carols.

The dinner turned out a success, and those of the committee who stayed to the meal were unanimous in praise of the pudding. Turkey, with the traditional cranberry sauce, the inevitable celery, sweet potatoes, and ice-cream, made up the menu; and, after the hungry had been satisfied, presents were exchanged, Christmas cards distributed, and admission of visitors to the patients was unlimited.

Later in the afternoon the big tree was dismantled, and a universal tea party, to which all and sundry were invited, proved a great success, the crowning point being the sampling of slices from a big Buzzard cake, which had been sent out to the matron. The Chairman and Treasurer, with that kindness and pleasant feeling which Americans usually show to the stranger within their cities, each sent a lovely basket of English flowers to the matron, no flower being allowed to figure in these posies but those of true British origin. So that this first Christmas in the United States was extremely happy. But I was obliged to promise the staff that our "elegant" Christmas should not detract from the observance, *à la* Stars and Stripes, of the New Year.

### Christmas at Marseilles.

### DISPENSAIRE DES ENFANTS MALADES.

(Contributed.)

Christmas Day is always a great fête day at this children's hospital, and it is all the more appreciated by the poor that next to no notice is taken of it in the town hospitals. An extra dish served at their mid-day meal is all the patients ever expect.

Here, at the dispensaire, on the contrary, the *fondatrice*, the Comtesse Gilbert de Voisins, endeavours to make of this fête a Christmas Day resembling an English one as far as bounty and rejoicings can do so.

None but the poorest being admitted, either to the wards or in the out-patient department, clothes are necessarily a very welcome gift. For days beforehand preparations are being made in the shape of bundles of clothes suited to the requirements of the family to which the little patients belong.

In the large waiting hall, nicely decorated, a fine tree is erected, surmounting the usual picturesque group of the Holy Nativity, and the artistic arrangement of Eastern scenery, over the hills and vales of which the Shepherds and Wise Men are wending their way. The consulting-rooms are turned to good use; in one a fine trade is being done in the clothes line, only to be compared to the rapid consumption of the substantial *goûter* provided in another, while from the *salle de pansements* lively strains are issuing, testifying to the musical talent and good nature of a surgeon.

The ward children are first brought down to enjoy the tree, their many presents, and the food provided, after which they are returned to their cots, and the doors are thrown open to the out-patients, impatiently waiting, tickets in hand. The noise and confusion, exclamations, clapping, all testify to their enjoyment and delight, and it requires all the help of those present, the friends of the *fondatrice*, doctors and nurses, to guide the excited little folk round, passing in good order to the clothes, tree, refreshments, and finally out by another door.

This fête has been regularly given for five years, in fact ever since the hospital was founded; every year the effort is made to give greater pleasure and relieve distress on as large a scale as means will permit. Great is the credit due to those who thus contribute yearly to this good cause, both in time and money, and thanks also for the many gifts which annually are sent by outsiders, and contribute largely towards making Christmas Day at the *Dispensaire* an eventful and happy day for many a poor little cripple.

### Christmas in the Fatherland.

By an English Nurse.

Germans are accounted stolid, yet they invest children and child-life with more poetry and romance than does any other nation. They are accredited with atheism, but it would be difficult to find, even in the devout Roman Catholics of Italy, a more reverent faith in the influence of the Christ-child at Christmas than exists in the heart of the phlegmatic Teuton. A German children's hospital is always charming, but at Christmas it is like a page out of Fairyland, everyone being agreed to banish on this one day of the year all prosaic fact, and to live in a world of fancy and illusion.

For weeks before the festival great preparations had been going on in the children's hospital where I was first introduced to the charming German fashion of keeping Christmas. Such a scrubbing and cleaning I had never before seen. Even the chimneys were swept, for, said the good sister to the wondering babies, "Santa Claus must never spoil his snowiness by coming down a dusty chimney."

The unpacking of the myriad parcels of toys and "goodies" which kept arriving gave the wards the appearance of a chronic condition of jumble sales. And the lovely evergreens and firs with which to decorate, came in such quantity as to create a fear lest all the forests of the country had been shorn ruthlessly to make one hospital beautiful. Spangles fashioned in every conceivable shape were so plentiful that a little *madchen* thought "all the stars had come down from heaven to visit us."

On Christmas Eve, when the beautiful decorations of holly and fir were complete and the morrow's feast of cakes and sweets had been displayed, Sister said, "We must not forget, my little ones, to share our good gifts." And she showed them some pots in which were set large boughs of fir, with great bunches of yellow oats tied among the fairy branches. "These," she said, "are for the birds, for they, too, must have a happy Christmas." And then she repeated that sweet old Norse rhyme:—

"Come hither, little birds, merry of mood,
By barn door and dwelling-house corn ears are strewed.
Christmas comes hither,
Then may ye gather
Food from the bread-giving straw golden-hued."

On Christmas morning one of the big doctors covered in white cotton wool with venerable beard, whose snowiness threw out the brightness of his rubicund countenance, came round in a wonderful chariot, whose construction had occupied so many hours, with jingling bells. "Hark, that's Santa Claus," said the children, when they heard the bells. The procession began at seven a.m., and a right royal progress King Christmas made on his car laden with toys for good and bad children alike. For even naughty little Hans, the pickle of the ward, whose heart misgave him as he recollected nurse had threatened to communicate his shortcomings to Father Claus, had a big bear put into his quaking hand.

It would be difficult to pen a picture of the glories of the car. Its framework was an old tricycle, but by the time shafts were added, a canopy erected, and huge quantities of spangled snow piled on, and fir branches festooned round, its commonplace identity was successfully concealed. So that its propulsion round the wards appeared quite magical in the eyes of the children. Later there was the big tree to dismantle, with wonderful presents cunningly hidden amongst the foliage, the branches being resplendent only with spangles and lights. The convalescents joined hands, and danced round the tree singing religious songs illustrated in pretty kindergarten fashion by action and expressive gesture. Then followed the singing of "Lieb Vaterland," for every little German is taught to be a citizen and a patriot before he can run alone.

Tired out with pleasure and excitement the children were soon ready for tea, with its array of cakes and sweets; a few more carols were sung, and a score of tired little heads were soon on their pillows living over in dreamland the events of the happiest Christmas I ever saw in a children's hospital.

### Christmas in Egypt.

By "Prowler."

Though nurses engaged in any of the Egyptian towns or provinces have none of the excitement and pleasures enjoyed by those at home at this season of the year, they have not such a dull time as one might be led to suppose, considering they are so far away and in a Mahommedan land. True, there are no wards to decorate, Christmas trees for the patients to be thought of, no carols to be sung, nor any of the little amusements and worries which are inevitable in the carrying out of these festivities at home. But English nurses will have their Christmas if it is by any means possible, and if, in Egypt, they take it rather solemnly, it is due more to their surrounding circumstances and the religion of the country than to anything else. And they enjoy the day as much in Egypt as they would elsewhere. On Christmas morning they have their little presents to open and admire, and twice during the last five years the mail has chanced to arrive on that day, making it very much more like home, with all the Christmas letters, cards, &c. Then, after a hurried breakfast, they go to the English church, which the English residents, assisted by "Tommy Atkins," always decorate most beautifully, and if there is an absence of ivy and holly, the lack of these is amply compensated for by an abundant supply of the most glorious roses, arum lilies, tall palms, and even violets. And the taste displayed and the effect produced would eclipse many a church at home. The service in Cairo is usually bright, and the church crowded.

Later on in the day there is the Christmas dinner, which includes roast turkey stuffed with apples and boiled chestnuts—a dish which the native cook really enjoys preparing, and does well—and plum pudding, the success of which, if less than that of the turkey, is more likely to be caused by over-anxiety on his part than want of interest. He cannot understand why a pudding should require six hours' boiling. If asked "Is the pudding all right?" he grins and says "Yes." You say "Is it boiling?" he replies "No, but it is in the pan!" But nurses who live in Egypt become accustomed to the cook's failures, and the pudding is probably there only because it is an English dish, and right for Christmas day—not necessarily to eat. There is always a plentiful supply of freshly gathered oranges, bananas, dates, and Turkish coffee to bring the menu to a close.

The climate at this season is perfect, just like July at home, with all its brightness and sunshine, and none of its sultry oppressiveness and rain. A curious contrast to the sort of weather we associate with Christmas in England!

### A Christmas Story.

"Nurse," said a weak little voice, "baint you coming to do em on Christmas Day?"

"If you want me I shall certainly come and put you comfortable."

Little Jack smiled.

"Why o' course I shall want you, ever so; but my mother was a-sayin' as most likely you'd think to go and 'joy yerself along of your own folks. P'raps yer aint got no folks?" he added enquiringly.

"Oh, yes, I have, Jackie, and to tell you the truth they scold me sometimes!"

"Do they?" asked the small patient. "I know jolly well as they wouldn't if *I* was near by."

"Well, Jackie, then I think I shall refer them to you next time they tell me that it is my duty to go to them at Christmas. You see people do not understand how badly sick folks want a nurse every day of the week."

"'Specially when they are so dreadful poor," added Jackie, with precocious insight into the sadder side of life.

"Mother," he shouted a minute later, "I say, Mother, ain't yer near done yer bit o' washing?"

In response to the call, a poorly clad, ill-nourished woman came in, wiping the soap suds from her bare arms.

"I shan't be above another half-hour, Jack. I thought as I'd get on a bit whiles the nurse was along of you. How's 'is back to-night, Nurse?"

"About the same, Mrs. Edwards; you must not expect to see a great deal of difference yet. The doctor seemed better pleased yesterday, so perhaps the new treatment is suiting him. He is a capital patient." While she spoke, nurse finished making the bed, and began arranging the things on the small table beside it.

"That's a mighty handy little table," remarked Mrs. Edwards; "Jackie is wonderful set up with you a lendin' it to him. He can 'elp 'isself to a drink of milk or a grape or two when I'm away, and it all helps to pass the time; don't it, Sonnie?"

Nurse was buttoning her cloak, and then she picked up her bag, the contents of which were as miscellaneous as they were useful.

"Now, Jackie, good-night, my dear." And the district nurse stepped out of the cottage into the early dusk of a December evening. The rain poured down on her serviceable umbrella with a steady thud, and she stepped into ruts and puddles as she traversed the ill-kept village street.

"How is your wife to-night, Morris?" she asked, as she reached a cottage standing back from the road and found a labourer awaiting her on the door-step.

"Well, nurse, the doctor was in a while back, an' he said as he'd a shade more hope of 'er. He went so far as to say that 'twas only good nursing 'as could 'ave pulled 'er through. 'She's not out of the wood yet,' he sez, as he got on 'is horse, 'but, please God, we'll 'ave 'er downstairs again in the beginning of the new year. Ask nurse to go on just the same till the end of the week.'"

Nurse laid her dripping cloak over the back of a kitchen chair, and then she held out her hand.

"Now, Morris, I must shake hands with you! That's the best bit of news I've heard to-day. The doctor is so careful that when he talks like that you may rest assured that the worst danger is over. I will go up to her at once."

"Stop a minute, nurse," said the man. "Would you mind telling me if you know anyone as I can get in for Christmas?"

"Do you mean that you are not satisfied with me?" and nurse turned back from the foot of the stairs.

The man hastened to reassure her.

"Why, nurse," he said, with a kind of smothered sob in his voice, "an angel out of heaven couldn't do more for us than wot you've done. You see, you're the first reg'lar clever, eddicated nurse as we've ever 'ad in the parish, an' we thought as you'd be like them 'alf-bred 'uns, as we was used to before."

"I must not stop talking any longer," said nurse, "or your wife will think I have forgotten her. Do you know you are the fourth person who has hurt my feelings to-day? Even my little Jackie took it into his silly little head to doubt me! When I think healthy, happy people need me more than you poor sick folks, *then* I'll run away from you at Christmas, but not before."

That winter was long remembered in the village as "that year as nurse spent her first Christmas with us, and, please God, He'll spare her to us for a many more." "We could do with a good few more women like her!" was the emphatic rejoinder of Jackie's careworn mother.

### Christmas Gifts.

CHRISTMAS shopping may be a pleasure, but to judge from the expression of its votaries, it is also a very severe task. If this is the case in London, where tempting shops with their suggestive and gaily-decked windows abound, what must it be for country givers, many of whom, like nurses for instance, have only a short leisure time at their disposal. It has been our custom each year to describe some of the Christmas novelties that have come in our way, and hence the following suggestions.

Messrs. Longmans' (39, Paternoster Row, E.C.) classified catalogues of books will prove a most invaluable aid to those who have decided to make a gift of books. It is only necessary to write "I desire a list of children's books, of scientific books, of fiction, &c.," and the post will bring just what we need.

Messrs. Dean & Co., 60A, Fleet Street, have made gifts for the little people their particular care. Their catalogue is a delightful study from its suggestiveness alone. Their productions are for real, veritable children; the matter and the illustrations are admirably chosen, and the nursery literature commences as low in price as one halfpenny per volume. "Living Strewelpeter" alone will form an inexhaustible fund of amusement in the nursery. The illustrations are brought into action by a cardboard pulley to each, and as the subject is one intensely interesting to the juvenile mind, namely, the adventurous career of a group of naughty children, dramatised by means of the pulley, the popularity of the book can hardly be over-estimated. There are some wonderfully-arranged A B C's on indestructible paper and otherwise, and for the older children Miss Maud Dean has compiled a neat little volume of verses for children's recitations, and so many other delightful novelties, that we can only refer our readers to the catalogue, or better still, to Messrs. Dean's emporium itself.

There are few amongst "grown-ups" to whom a present of a bottle of Eau de Cologne comes amiss, and how welcome is it in the sick-room. We have more than once stated that Messrs. Muhlen's 4711 Eau de Cologne is the very best we know, and we have often heard our opinion confirmed. All their scents are excellent in quality; the Rhine violets and Marechal Niel varieties are especially delicious. These scents are utilised to perfume the no less excellent soaps, which can be had under the name of "visitors' soap" in small tablets, a very convenient form. We have heard it whispered that the Emperor of Germany himself patronised a beautiful rose scented soap of Messrs. Muhlen's manufacture. These specialities can be had suitably put up in ornamental bottles and boxes.

One of the most useful forms of literature to country cousins as Christmas draws nigh, is the modern innovation, the illustrated catalogue, and amongst these handbooks we know none more serviceable than that issued by Messrs. Mappin and Webb, which they are pleased to send on application. Much more suggestive than any words of ours are the neatly illustrated pages, numbering nearly 60, which inspire the imagination with gifts from one shilling to one hundred pounds, fit for rich and poor, old and young.

Amongst useful and comfortable gifts suitable for the season, we are constrained to mention the neat little black knitted shoulder capes, made by the Nottingham Knitted Corset Company. Something similar, more suited for youthful and evening wear, we have seen at Messrs. Owens, in Westbourne Grove. In this same land of shops, at Messrs. Hanneys, are to be found the most remarkably cheap series of books that we have yet met with. It is difficult to understand how high class literature, neatly bound, can be produced, as it is by this firm, at 6¾d. the volume. The series comprises a number of Dickens's works, and others by well-known authors.

The Oxford University Press have brought out another of their delightful little thumb edition volumes this Christmas. This time they have selected the "Pilgrim's Progress," which is a most suitable addition to the series, and forms a charming little present.

For the poor we would remind our readers that flannelette blankets under three shillings a pair form useful gifts.

How difficult it is to find presents to please the taste of our male friends! The field of their wants is so limited. One thing we may be sure of—our medical friends must start new visiting lists with the new year, and there is a useful offering. Messrs. Wright, of Bristol, whose agents are Messrs. Simpkin, Marshall, and Co., make these lists a speciality, and we cannot do better than consult their list. Three varieties are to be had. The Improved List, form A, only requires writing once a month. The Improved Perpetual List may be commenced at any time, and the Improved Weekly List, which is after the old form, can be had should this be preferred. All the books contain useful general and professional information, and special tables and arrangements for recording facts to be remembered. A good pencil is added, and the bindings are strong and neat. If our doctor friend is already supplied with his list, a calendar from Marcus Ward, handsomely bound in leather, for one shilling, will usefully find a place on the writing table. For non-professional friends the Engagement Calendar, price one shilling, or the Concise Diary, beautifully bound in Russia leather, for five shillings and threepence, produced by the same firm, add to the selection. Dean and Son, of 160A, Fleet Street, publish a fascinating little diary called Debrett's Waistcoat Pocket Diary.

And now for a word about Christmas cards, calenders, and the like. We cannot do better than recommend Christmas purchasers to secure some of Messrs. Marcus Ward's fascinating productions. We had occasion to speak in praise of these last year, and can now not only heartily reiterate all we said then, but also bear testimony to the fact that in variety and uniqueness of design this year the firm have surpassed themselves. Firstly, in the matter of cards, we would draw attention to the wonderful shilling box containing fifty cards, which are really of excellent value. What an amount of time and trouble is saved by thus investing a modest shilling so advantageously! For those who attach importance to the personal selection of their Christmas cards, there are all sorts of cards at all manner of prices. The shilling series are beautifully printed on artistic paper with charmingly engraved frontispiece—the whole tied together with a dainty riband. A really delightful and artistic gift is to be found in "Watchman, What of the Night?" a Christmas carol, the words of Henry Smith, set to music, and printed and illustrated in black and red on water-colour paper. We cannot understand how it has been possible to produce so handsome a piece of work for one shilling. At the same price are well-executed engravings of graceful female figures, little booklets, and many other varieties of novelties in Christmas greetings, and for smaller sums, down to one penny each, an equal variety is offered. We would especially notice the tribute to Scotch taste in the little tartan cards, enclosing appropriate verses. Turning to calendars, our task in selecting for special mention out of so charming a variety is by no means easy. In this loyal year of grace the calendar depicting Her Majesty the Queen and the Royal Family must, of course, have first place. The portraits are mostly very good. Red-letter Days, Modern Poets, Fair Women, Selections from Celebrated Authors, and many other interesting topics are chosen as subjects for other calendars sold for one shilling. "Our Native Land," with its frontispiece of Windsor Castle, should prove one of the most popular calendars of the season.

### Christmas Books.

METAPHORICALLY, it is the New Year which is described as the time for turning over new leaves, but putting metaphor aside, it is Christmas time which is the season of seasons for turning new pages, albeit those pages are of a literary character. With the days at their shortest, and the curtains drawn early on the outside world, Christmas lends itself as no other time does to the companionship of books, and as the demand so is the supply. Verily, there is no dearth of material; they come, they come! may fairly be said of this year's publications. The picture-book, the fairy story, the work of romance, written each respectively for the babies, the children, and the grown-up children of the world. Before us for review lies a pile of goodly books for readers of all ages and all kinds, varying with the needs of the public to whom they are severally dedicated. The child has its fairies; the boy his blood and thunder, his mighty heroes, and his stamp book; the "grown-ups" their fairy realm, too, of a later date! And having classified thus generally let us discuss individually some very charming Christmas books, to which we will call our readers' attention, beginning with that elementary stage of fictional art, the picture book. In the matter of these our little ones are well off this year. What more can the heart of child desire than "The Rainy Day Picture Book"* and "Some More Nonsense for the Same Bodies as Before, written and illustrated by a Nobody."† The first of these is a daintily got up collection of prints, with accompanying and descriptive letterpress, whilst the subject-matter of the second-mentioned book is epitomised in its title. Here are many clever sketches, printed in colour on thick, untearable paper (a wise forethought against baby fingers), and a collection of quaintly imaginative jumble verses. A certain well-known physician keeps a copy of this book on his consulting-room table as an antidote to his patients' dismal reflections on their physical ailments. From picture books, on which we fain would linger longer, we turn to fairy stories. If this old world of ours is daily becoming more material, it is not evinced in this department. There are more fairy stories than ever this season. And by fairy stories we mean genuine works of the imagination. Of course, these books are written for the reader to whom pixies and hobgobblins are something other than creations of the human brain. But the hands of Time work backward, and grown-up children once more regain the faiths of their childhood, clothe the holly-berry trees with dwarf personages, and invade the glowing embers with inhabitants after wandering through this realm of Fairyland Stories. "Prince Boo Hoo and Little Smuts"‡ we predict will gain the kingship among juvenile literature this Christmas. "Prince Boo Hoo" is written by the Rev. Harry Jones, and drawn by Gordon Browne, R.I., and so well drawn, too, by both his portrait painters that it is an open question to whom he most will owe his popularity. For, of course, never for a second do we doubt the actuality of this young man's existence. His history is one of adventure. Of his circumstances we are told: "Prince Boo Hoo was the only son of King Starzungartnoz and Queen Kizzimforwolteveredid. So you may be sure he was a great favourite with the Court and Nation." We are first introduced to him "one Tuesday morning, when he was playing in the Queen's dressing-room and cutting Acts of Parliament to pieces with a pair of gold scissors." When he commenced clipping at a Reform Bill his royal father made his entrance, with his slippers on, and his crown awry. A bad sign this: and the King hastened to explain his displeasure at certain conduct on the royal heir's part, who, it appeared, had peremptorily taken it into his hands to dismiss the Court gardener "because strawberries would not grow in winter." Upon this most justifiable explanation the Sovereign parent rang the bell and ordered "all the gardeners' heads to be cut off as soon as they had sent in the vegetables for dinner." And in the same humorous strain the story continues after the entrance of a fairy Godmother of Prince Boo Hoo. "Little Smuts" follows next in these pages, and the illustration shows her encounter with a figure in the fire, who materializes, whilst she is gazing one day into the embers. "Little Smuts'" subsequent adventures with this strange creature are little less marvellous than are those of her fictional predecessor, "Alice in Wonderland." "School in Fairyland,"* as its title describes it, is "a school where little mortals went to be taught by fairies." Mrs. Strain is a well-known writer of juvenile books, and her latest contribution is profusely illustrated. "The Green Garland"† and "King Pippin"‡ are neither of them fairy stories, but are suitable for children who have grown out of the age when fiction is necessarily centred round the unreal. Indeed, the charm of the latter depends to a great extent on its delightfully "real" tone; it strikes one as having been drawn from little people, who were real little people. It is the story of a boy and girl who were twins, and is written autobiographically by the latter, "King Pippin," as he is called.

"The Green Garland," after which the book takes its name, is a symbolical name for "the one great deed that stands waiting for every man in every station," and after which King Pippin describes the search, telling the story with a quaint and ready humour, and with a pathos, too, perhaps, somewhat improbable considering the describer's tender years. Any book feigning to be written by a child runs this risk of improbability when we compare it in our thoughts with the traditional inconsequence of the youthful mind. Of "Sydney's Chums"‖ much could be said in its praise would space admit. It is more carefully worked out, perhaps, than any volume we have so far concerned ourselves with in this review. Sydney, the hero of Miss H. F. Gethen's book, is a precocious boy, whose great expectations give him a fictitious value in the eyes of a dull old guardian, who keeps him metaphorically in a glass case, safe from the contamination of child companions. Circumstances arise, however, which lead to an extension of Sydney's acquaintance with his fellow creatures, and several "chums" of a heterogeneous order play a significant part in the little boy's life. The writer has her subject well in hand. The tale, part of which concerns itself with an East-end alley, is undoubtedly drawn from life, and the whole *mise en scène* of the young Sydney's career has a certain distinction of its own, and does credit to the authoress.

* "The Rainy Day Picture Book." (London: Ward, Lock, and Co.)
† "Some More Nonsense for the Same Bodies as Before, written and illustrated by a Nobody." (London: Gardner, Darton, and Co.)
‡ "Prince Boo Hoo and Little Smuts." Harry Jones. (London: Gardner, Darton, and Co.)

* "School in Fairyland." Mrs. Strain. (London: T. Fisher Unwin, Paternoster Buildings.)
† "The Green Garland." Francis G. Crompton (London: A. D. Innes and Co.)
‡ "King Pippin." Mrs. Gerard Ford. (London: Jarrold and Sons.)
‖ "Sydney's Chums." H. F. Gethen. (London: Blackie and Sons.)

### Everybody's Opinion.

### HELP WANTED.

NURSE ADELAIDE BOURNE, Union Infirmary, Wilton, near Salisbury, writes: I am nursing for the Workhouse Infirmary Association at this infirmary. My cases are mostly the old and infirm, both men and women, some of whom are not able to leave their wards and have not done so for years. I have been speaking to them about Christmas decorations, and they said how much they should like to have a Christmas tree and have the children in. I thought, how could I give them that pleasure, then it struck me if I wrote and asked you perhaps you would make an appeal in THE HOSPITAL for gifts for me. There are fifteen children, ages from one year to twelve years; fourteen old men and fourteen old women. Toys, tea, pipes, anything in the shape of gifts would be thankfully received by me. Please pardon my troubling you, but I should be so glad if I could give my poor people this pleasure this Christmas.

### Help the Nurses to Help the Sick.

**Royal National Pension Fund for Nurses,** 28, Finsbury Pavement, E.C.—This Fund has during the past year maintained the uninterrupted success which has attended it ever since its establishment. In spite of the great attention drawn to the Fund by H.R.H. the Princess of Wales' reception in 1895, and the natural increase in the number of nurses joining caused thereby, the recruits to the Pension Fund in 1896 will hardly fall short of the previous year, which up till then was the record year of the Fund, for close on 800 nurses have been enrolled this year. Over £1,000 has been distributed in sick pay, a fact which speaks eloquently as to the blessing which this branch of the Fund's work must be to nurses, more especially, of course, to those working on their own account. The most notable event in connection with the Fund is the spontaneous endeavour now being made by the policyholders to double the capital amount of the Junius S. Morgan Benevolent Fund, for it has been found by the committee of that fund that its scope of benevolence is sorely hampered by want of means.

**The Junius S. Morgan Benevolent Fund** is an auxiliary to the above fund for nurses, and was founded through generous contributions from nurses themselves, and raised to handsome proportions by the munificence of the Morgan family and many other friends to nurses. The work is done by volunteers, under the supervision of an influential committee, which devotes time and care to the investigation of claims and the relief of urgent cases. Hon. Secretary, Miss Rosalind Pritchard.

**East London Nursing Society.**—The object of this society is to nurse the sick poor of East London in their own homes by means of trained resident nurses, each nurse living in the parish in which she works. The extent of the society's useful work is shown by the fact that in 1895 the staff of 33 nurses attended to 5,438 persons, to whom 130,483 visits were made. Annual subscriptions and donations to the general fund are asked for. Secretary, Mr. Arthur W. Lacey, 49, Philpot Street, Commercial Road, E.

**Metropolitan Nursing Association,** Bloomsbury Square, W.C.—Founded in 1875 as a Training School and Home for District Nurses who have previously gone through a full course of hospital training, and who nurse the sick poor in their own homes within a radius of a mile and a half from Bloomsbury Square. This Association is now the central training home for the Queen's Nurses. Superintendent, Miss Gray.

**Queen Victoria's Jubilee Institute for Nurses.** Offices at St. Katharine's Hospital, Gloucester Gate, Regent's Park, N.W.—The institute trains nurses in district work, and supplies nurses trained in district as well as hospital nursing for the sick poor in their own homes. Applications for information should be addressed to Miss Peter, the Inspector. Scotch, Irish, and Welsh branches also exist, and numerous associations have affiliated themselves with the institute.

**Up-Country Nursing Association for Europeans in India.**—The chief object of this association is the provision of skilled nursing for Europeans, especially civilians, in the up-country districts of India. The association in London selects the nurses, and pays all their expenses until they arrive at their destination in India. Once established there, the cost of maintaining the nurses falls upon the Local Committee. The chief difficulty which the Local Committee has to meet is that caused by the frequent changes of place of employment of the subscribers. The fees earned by the nurses are not sufficient to cover all expenses, and must be supplemented by subscriptions. The expenses in England are entirely met by voluntary donations and subscriptions. No salaries or commissions of any kind are paid in England, and accounts of all expenditure are published. Hon. Secretary, Major-General J. Bonus, R.E., The Cedars, Strawberry Hill.

**Workhouse Infirmary Nursing Association.** Offices, 6, Adam Street, Strand, W.C.—The objects of this association are: To raise the standard of public opinion on the whole question of workhouse nursing; to secure the appointment of trained ladies as matrons in all separate infirmaries; and to train and supply nurses to workhouse infirmaries in London and the provinces. The association has trained altogether 267 probationers, and 120 nurses are now at work. Increased financial support is urgently pleaded for, to extend the work of training probationers, to which purpose the greater part of the funds are applied. Subscriptions will be gladly received by the Hon. Secretary, Miss Wilson, 13, Barkston Mansions, S.W.

**Invalid Children's Aid Association.**—The object of the association is to help invalid and crippled children in their own homes. A system of regular visiting is organized and the ladies who volunteer for this work find out what is most needed by the little people, and endeavour to supply it. Spinal carriages, perambulators, surgical instruments, and bed linen are lent; clothes, toys, picture books, &c., are given; and tickets for seaside and country convalescent homes are distributed. Funds are greatly needed by the committee to enable them to carry on their work. Subscriptions will be gladly received by the Hon. Secretary, Mr. Allen Graham, 18, Buckingham Street, Strand, W.C.

**Irish Distressed Ladies' Fund,** 17, North Audley Street, W. Patron, H. M. the Queen. Relief is given independently of any question of politics or religion; employment is found, when possible, for those able to work; pecuniary help is given to the aged and infirm; and the education of children is paid for. The expenditure in 1895 amounted to £2,954, against which only £1,626 was received, so that the Fund is in immediate want of £1,500 to clear off this debt. Secretary, General W. M. Lees.

**"The Hospital" Convalescent Fund.**—Since the first establishment of a nurse's free bed many tired and delicate workers have enjoyed a much-needed change of air such as they could not possibly have secured for themselves without help. Experience has proved that it is better to let the nurses have a choice of locality rather than to send them to one settled place, and therefore the title of THE HOSPITAL Convalescent Fund has replaced the "Nurses' Bed." The object is, however, the same, namely, the provision of rest for weary workers, amidst suitable surroundings, without any of that anxiety about ways and means which retards convalescence. The fund is a small one, and contributions which would increase its field of usefulness are invited by the Hon. Secretaries, care of the Editor of THE HOSPITAL.

### Novelties for Nurses.

NEW DESIGNS AT MR. FREDERICK PENBERTHY'S.

Very tempting at the present time are the windows of 390, Oxford Street, where Mr. F. Penberthy, Court glover and hosier, is displaying an unusually attractive collection of seasonable garments, useful underclothing, dainty lingerie, and other elegant novelties. It is, however, with the combination garment, to which is attached a cholera belt, that we are principally concerned. This delightful contrivance obviates the necessity of wearing a separate belt, which has a disagreeable tendency to ruck up in an unsightly manner after it has been worn for a short time. Here we have a perfectly-fitting garment with a double thickness of shaped material going round the abdomen and loins and no possibility of its moving out of place. The comfort and convenience of this arrangement is obvious, and only requires to be known to become deservedly popular. These combinations are made in white or natural wool, the added belt being of either pink or blue, firmly stitched on top and bottom. Another delightful novelty is a loosely knitted woollen night vest in soft Berlin wool. It can be had in either white or pale pink for the modest sum of 1s. 6d., and is superior to the ordinary gauze vest by reason of its greater elasticity and porosity. Night-dresses in natural, white, and pink sanitary wool had a most comfortable appearance, and would be ideal garments for those who feel the cold or suffer from rheumatism. There is also a good selection of stockings in the new sanitary black, which are very reasonable in price and of excellent quality.

